# Holistic AI analysis of hybrid cardiac perfusion images for mortality prediction

**DOI:** 10.1038/s41746-025-01526-0

**Published:** 2025-03-13

**Authors:** Anna M. Marcinkiewicz, Wenhao Zhang, Aakash Shanbhag, Robert J. H. Miller, Mark Lemley, Giselle Ramirez, Mikolaj Buchwald, Aditya Killekar, Paul B. Kavanagh, Attila Feher, Edward J. Miller, Andrew J. Einstein, Terrence D. Ruddy, Joanna X. Liang, Valerie Builoff, David Ouyang, Daniel S. Berman, Damini Dey, Piotr J. Slomka

**Affiliations:** 1 Departments of Medicine (Division of Artificial Intelligence in Medicine), Imaging and Biomedical Sciences, Cedars-Sinai Medical Center, Los Angeles, CA USA; 2 Center of Radiological Diagnostics, National Medical Institute of the Ministry of the Interior and Administration, Warsaw, Poland; 3 Signal and Image Processing Institute, Ming Hsieh Department of Electrical and Computer Engineering, University of Southern California, Los Angeles, CA USA; 4 Department of Cardiac Sciences, University of Calgary, Calgary, AB Canada; 5 Section of Cardiovascular Medicine, Department of Internal Medicine, Yale University School of Medicine, New Haven, CT USA; 6 Division of Cardiology, Department of Medicine, and Department of Radiology, Columbia University Irving Medical Center and New York-Presbyterian Hospital, New York, NY USA; 7 Division of Cardiology, University of Ottawa Heart Institute, Ottawa, ON Canada

**Keywords:** Radionuclide imaging, Tomography, Cardiovascular diseases

## Abstract

Low-dose computed tomography attenuation correction (CTAC) scans are used in hybrid myocardial perfusion imaging (MPI) for attenuation correction and coronary calcium scoring, and contain additional anatomic and pathologic information not utilized in clinical assessment. We seek to uncover the full potential of these scans utilizing a holistic artificial intelligence (AI) approach. A multi-structure model segmented 33 structures and quantified 15 radiomics features in each organ in 10,480 patients from 4 sites. Coronary calcium and epicardial fat measures were obtained from separate AI models. The area under the receiver-operating characteristic curves (AUC) for all-cause mortality prediction of the model utilizing MPI, CT, stress test, and clinical features was 0.80 (95% confidence interval [0.74–0.87]), which was higher than for coronary calcium (0.64 [0.57–0.71]) or perfusion (0.62 [0.55–0.70]), with *p* < 0.001 for both. A comprehensive multimodality approach can significantly improve mortality prediction compared to MPI information alone in patients undergoing hybrid MPI.

## Introduction

Myocardial perfusion scintigraphy is widely used for the evaluation of coronary artery disease (CAD), with over 15–20 million scans performed worldwide^1,2^. During myocardial perfusion imaging (MPI), a low-dose non-contrast computed tomography attenuation correction (CTAC) scan is often used to correct for soft-tissue attenuation, leading to improved diagnostic accuracy^3,4^. Attenuation correction by computed tomography (CT) is recommended by American Society of Nuclear Cardiology guidelines^5^. Although the myocardium is the structure of principal interest during SPECT/CT MPI, its CTAC scan provides a wealth of additional information about other visible organs. Incidental findings have been reported in up to 59.5% of SPECT/CT MPI studies, of which some are clinically important and necessitate further diagnosis and treatment^6,7^.

However, due to limitations in the quality of CTAC images (low dose, no electrocardiographic gating), detection and characterization of abnormal findings on CTAC can be challenging^8^. Consequently, the additional information present in hybrid cardiac scans is often underutilized during clinical reporting. While some methods have been developed to derive information about coronary artery calcium (CAC) and epicardial adipose tissue (EAT) from CTAC scans^9,10^, many other potentially clinically important features, like extracardiac structures, are present in these scans, yet to date their added value to MPI has not been systematically evaluated (Supplementary Table 1).

The aim of this study is to develop a holistic artificial intelligence (AI)-based approach for the prediction of all-cause mortality from SPECT/CT MPI utilizing all possible information contained in the hybrid images and to separately evaluate the value of CTAC images for this purpose, which have been previously underutilized.

## Results

### Patient Characteristics

**Fig. 1:**
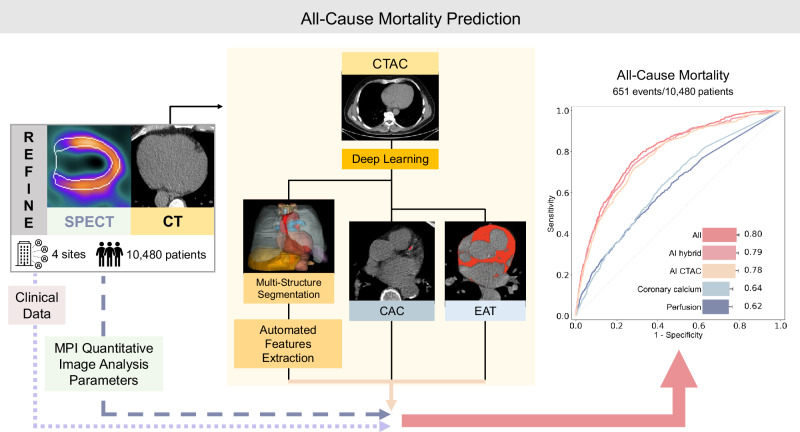
Central illustration. Artificial intelligence (AI) model integrating fully automated multi-structure computed tomography attenuation correction (CTAC) segmentation, quantitative image analysis (radiomics), deep learning (DL)-based coronary artery calcium (CAC), and epicardial adipose tissue (EAT) in all patients undergoing myocardial perfusion imaging (MPI) single-photon emission computed tomography/computed tomography (SPECT/CT). Receiver-operating characteristics curve for all-cause mortality and area under the receiver-operating characteristic curve values of Coronary calcium (DL-CAC score), Perfusion (stress TPD), the AI CTAC model (including DL-CAC, DL-EAT, and radiomics), the AI hybrid model (combing the CTAC model with stress MPI quantitative image parameters and stress variables) and the All model (incorporating AI hybrid image features, and clinical data).

In total 10,983 participants from 4 sites were enrolled in the REFINE SPECT registry, of which 500 CTAC scans from one site were used for EAT-model training and validation. Of the 10,483 remaining participants, 3 were excluded due to incomplete CTAC scans. The final study cohort consisted of 10,480 participants (Fig. 1, Supplementary Fig. 1).

Table 1 represents baseline characteristics stratified by sex. Of all participants, 5745 (54.8%) were male, and median age was 65 with an interquartile range (IQR) of (57, 73) years. During the median 2.9-year (IQR 1.6–4.0) follow-up period, 651 (6.2%) patients died. Table 2 shows baseline characteristics stratified by ACM. Normal myocardial perfusion was present in 7329 (69.9%) patients, of whom 345 (4.7%) died. Patients with normal perfusion were significantly younger (*p* < 0.001), more often female, and less often diagnosed with hypertension (*p* < 0.001), diabetes (*p* < 0.001), and dyslipidemia (*p* < 0.001) (Supplementary Table 2).

**Table 1:** Baseline characteristics for all participants stratified by sex

	All Participants	Male	Female	*P*-value
*N* (%)	10480	5745 (54.8)	4735 (45.2)	
Age [years]	65 (57, 73)	64 (56, 72)	66 (57, 74)	<0.001
BMI [kg/m^2^]	29 (25, 33)	28 (25, 33)	29 (25, 34)	0.019
Hypertension	6175 (58.9)	3371 (58.7)	2804 (59.2)	0.589
Diabetes mellitus	2684 (25.6)	1539 (26.8)	1145 (24.2)	0.003
Dyslipidemia	5085 (48.5)	2984 (51.9)	2101 (44.4)	<0.001
Smoking	1987 (19.0)	1224 (21.3)	763 (16.1)	<0.001
Family history of CAD	2771 (26.5)	1393 (24.3)	1378 (29.1)	<0.001
Prior CAD				
Prior Myocardial Infarction	750 (7.2)	522 (9.1)	228 (4.8)	<0.001
Past PCI	1508 (14.4)	1111 (19.3)	397 (8.4)	<0.001
Past CABG	636 (6.1)	506 (8.8)	130 (2.7)	<0.001
Mortality	651 (6.2)	398 (6.9)	253 (5.3)	<0.001
CT Quantitative Image Analysis Parameters				
DL CAC score	56 (0, 709)	171 (0, 1,184)	12 (0, 248)	<0.001
DL EAT volume [mL]	130 (90, 183)	143 (99, 198)	119 (83, 163)	<0.001
DL EAT density [HU]	−65 (−70, −61)	−65 (−70, −61)	−65 (−70, −61)	0.004
MPI Acquisition Parameters				
Stress Test Type				<0.001
Exercise	4732 (45.2)	2843 (49.5)	1889 (39.9)	
Pharmacological	5748 (54.8)	2897 (27.6)	2851(27.2)	
Peak Stress Heart Rate	112 (89, 146)	115 (88, 146)	110 (91, 142)	0.547
Peak Stress Systolic Blood Pressure	148 (128, 170)	150 (128, 172)	145 (126, 166)	<0.001
Peak Stress Diastolic Blood Pressure	80 (70, 86)	80 (70, 88)	80 (70, 85)	<0.001
ECG Response to Stress				<0.001
Negative	8010 (77.0)	4295 (75.0)	3715 (79.0)	
Positive	1167 (11.1)	708 (12.3)	459 (9.7)	
Equivocal	455 (4.3)	219 (3.8)	236 (5.0)	
Nondiagnostic	824 (7.9)	512 (8.9)	312 (6.6)	
Borderline	10 ( < 0.1)	4 ( < 0.1)	6 (0.1)	
MPI quantitative image analysis parameters				
Stress ejection fraction	64 (55, 72)	59 (51, 66)	70 (63, 77)	<0.001
Stress end diastolic volume	84 (64, 111)	102 (82, 127)	66 (54, 82)	<0.001
Stress shape index end Diastolic	0.58 (0.54, 0.62)	0.57 (0.53, 0.62)	0.58 (0.54, 0.63)	<0.001
Stress total perfusion deficit	2.6 (0.9, 6.0)	2.7 (1.0, 6.6)	2.5 (0.7, 5.5)	<0.001

Values are presented as *N* (%) or median (IQ1, IQ3).

*BMI* body mass index, *CABG* coronary artery bypass graft, *CAC* coronary artery calcium, *CAD* coronary artery disease, *CT* computed tomography, *DL* deep learning, *EAT* epicardial adipose tissue, *ECG* electrocardiogram, *HU* Hounsfield units, *MPI* myocardial perfusion imaging, *N* number of patients, *PCI* percutaneous coronary intervention.

**Table 2:** Baseline characteristics for all participants stratified by all-cause mortality (ACM)

	ACM	No-ACM	*P*-value
*N* (%)	651 (6.2)	9829 (93.8)	
Age [years]	71 (63, 79)	64 (56, 73)	<0.001
Male	398 (61.1)	5347 (54.4)	<0.001
BMI [kg/m^2^]	27 (24, 32)	29 (25, 33)	<0.001
Hypertension	417 (64.1)	5758 (58.6)	0.007
Diabetes mellitus	230 (35.3)	2454 (25.0)	<0.001
Dyslipidemia	329 (50.5)	4756 (48.4)	0.307
Smoking	125 (19.2)	1862 (18.9)	0.913
Family history of CAD	135 (20.7)	2636 (26.8)	<0.001
Prior CAD			
Prior Myocardial Infarction	69 (10.6)	681 (6.9)	<0.001
Past PCI	126 (19.4)	1382 (14.1)	<0.001
Past CABG	84 (12.9)	552 (5.6)	<0.001
CT Quantitative Image Analysis Parameters			
DL CAC score	353 (25, 1718)	48 (0, 652)	<0.001
DL EAT volume [mL]	143 (93,198)	130 (90, 182)	0.003
DL EAT density [HU]	−65 (−69, −60)	−65 (−70, −61)	<0.001
MPI Acquisition Parameters			
Stress Test Type			<0.001
Exercise	156 (24.0)	4576 (46.6)	
Pharmacological	495 (76.0)	5253 (53.4)	
Peak Stress Heart Rate	93 (79, 116)	114 (90, 146)	<0.001
Peak stress systolic blood pressure	130 (114, 150)	149 (128, 170)	<0.001
Peak stress diastolic blood pressure	70 (62, 80)	80 (70, 87)	<0.001
ECG response to stress			<0.001
Negative	518 (79.6)	7492 (76.2)	
Positive	52 (8.0)	1115 (11.3)	
Equivocal	11 (1.7)	444 (4.5)	
Nondiagnostic	69 (10.6)	755 (7.9)	
Borderline	1 (0.2)	9 ( < 0.1)	
MPI quantitative image analysis parameters			
Stress ejection fraction	58 (45, 68)	64 (56, 72)	<0.001
Stress end diastolic volume	93 (70, 130)	83 (64, 110)	<0.001
Stress shape index end diastolic	0.60 (0.56, 0.65)	0.58 (0.53, 0.62)	<0.001
Stress total perfusion deficit	4.4 (1.7, 9.9)	2.5 (0.8, 5.8)	<0.001

Values are presented as *N* (%) or median (IQ1, IQ3).

*BMI* body mass index, *CABG* coronary artery bypass graft, *CAC* coronary artery calcium, *CAD* coronary artery disease, *CT* computed tomography, *DL* deep learning, *EAT* epicardial adipose tissue; *ECG* electrocardiogram, *HU* Hounsfield units, *MPI* myocardial perfusion imaging, *PCI* percutaneous coronary intervention.

### Myocardial Imaging Perfusion Quantitative Image Analysis Parameters

In all patients, the median TPD was 2.6% (0.9–6.0) and was higher in male than female patients (2.7 vs. 2.5, respectively, *p* < 0.001) (Table 1). Significantly lower stress ejection fraction was observed in men compared with women (59% vs. 70%, respectively, *p* < 0.001). The median TPD in patients with abnormal perfusion was 8.9 (6.5, 14.2), whereas the median stress ejection fraction in this group was 57 (46, 67) (Supplementary Table 2).

### Coronary artery calcium and epicardial adipose tissue

CAC was 0 in 3,753 (35.8%) patients, >0–100 in 1982 (18.9%), >100–400 in 1462 (14.0%), and >400 in 3283 (31.3%) subjects. The median EAT volume and density were 130 mL (90, 183) and −65 HU (−70, −61), respectively (Table 1).

In patients with normal perfusion, 2903 (39.6%) subjects had no CAC, 1515 (20.7%) had CAC > 0 and ≤100, 1029 (14.0%) had CAC > 100 and ≤400, and 1882 (25.7%) had CAC > 400. The median EAT volume and density in patients with normal perfusion were 129 mL (89, 181) and −65 HU (−70, −61), respectively (Supplementary Table 2).

### Model performance

**Fig. 2:**
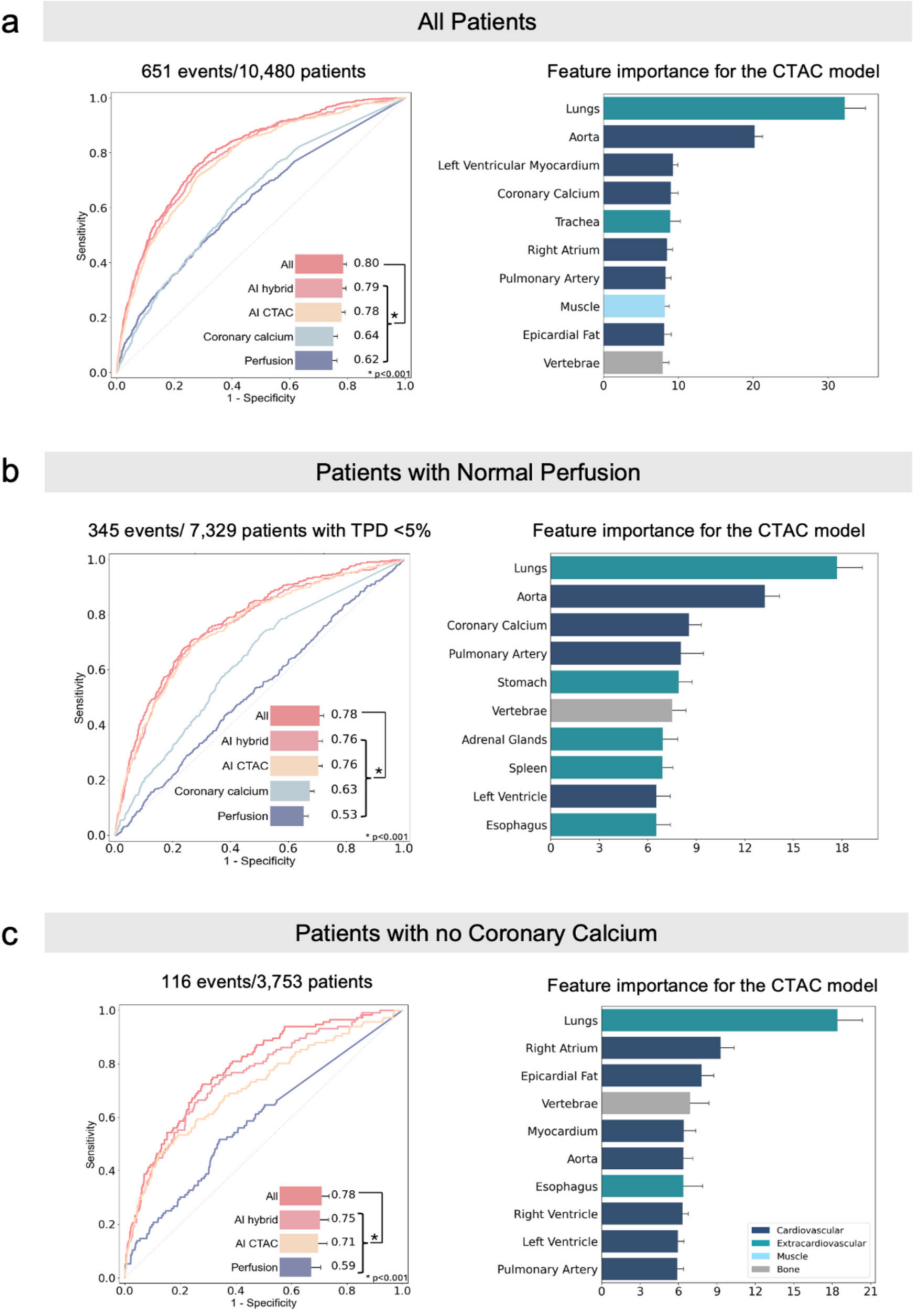
Model performance and feature importance scores for all-cause mortality. The performance of the model and feature importance scores were assessed (**a**) in all patients, **b** in patients with normal perfusion, and (**c**) patients with no coronary artery calcification. Normal myocardial perfusion was defined as total perfusion deficit (TPD) < 5%. Receiver operating characteristic curve for the artificial intelligence (AI) computed tomography attenuation correction (CTAC) model, including deep-learning (DL) coronary calcium, DL-epicardial adipose tissue (EAT), and radiomics, the AI hybrid model incorporating CTAC and myocardial perfusion imaging (MPI) data (stress MPI quantitative image parameters, Coronary Calcium [DL-coronary artery calcium score], Perfusion [stress TPD]), and a model combining CTAC, MPI, and clinical data (All). In all patients, the performance of the EAT model (not shown in the figure) alone was AUC 0.56, in patients with TPD < 5% AUC 0.54, whereas in subjects with no coronary calcium AUC 0.59. Feature importance score plot represents 10 segmented structures with the highest scores for the CTAC model.

Figure 2 represents the model performance and feature importance for mortality in all patients, subjects with normal perfusion, and patients without calcified lesions in coronary arteries. The lungs were the top feature in all patients, in patients with normal perfusion as well as in subjects without coronary calcifications. Supplementary Fig. 2 shows feature importance plots stratified by different sites and image quality. For all AI models in all patients included in the study, AUCs with 95% confidence interval (CI) are shown in Supplementary Table 3. There was a better performance of the AI CTAC model (AUC 0.78, 95% CI 0.71–0.85) than the EAT model (AUC 0.56, 95% CI 0.49–0.63, *p* < 0.001), and coronary calcium (AUC 0.64, 95% CI 0.57–0.71, *p* < 0.001) alone. There was a small but statistically significant difference in the prediction performance of the AI hybrid model and the CTAC model (AUC 0.79 vs. 0.78, *p* < 0.001). Additionally, the AI CTAC model outperformed the AI SPECT model (AUC 0.78 vs 0.65, *p* < 0.001).

AUCs with 95% CI for all AI models in patients with normal myocardial perfusion are shown in Supplementary Table 4, whereas in subjects with no coronary calcium in Supplementary Table 5. In the group with normal perfusion, the performance of the AI CTAC model was significantly better compared to Perfusion (AUC 0.76 vs. 0.53, respectively, *p* < 0.001). The AI hybrid model incorporating CTAC and MPI features had similar prediction performance compared to the AI CTAC-only model (AUC 0.76 vs. 0.76, respectively, *p* = 0.384). Among the patients with no calcium, the AI CTAC model significantly outperformed Perfusion (AUC 0.71 vs. 0.59, respectively, *p* < 0.001). The AI hybrid model was significantly better than AI CTAC-only model (AUC 0.75 vs 0.71, respectively, *p* < 0.001). Models were also evaluated across different sites and acquisition protocols, as shown in Supplementary Table 6. The AI model demonstrated consistent performance regardless of the acquisition protocols. However, Columbia and Ottawa showed significantly lower performance compared to Yale (Supplementary Table 6).

A subgroup analysis was performed using the best model (All model) across the following categories: white race, black race, female, male, older ( ≥ 65 years), and younger ( < 65 years). Due to limited data for other racial groups, the race-based subgroup analysis was restricted to black and white individuals. Our findings indicate that the All model demonstrates comparable performance across both male and female groups (AUC: 0.77 vs 0.79, *p* = 0.08) in Supplementary Fig. 3. Furthermore, the model exhibited better performance in individuals aged <65 years compared to those aged ≥65 years (AUC: 0.79 vs 0.74, *p* = 0.16). The difference between for the Black group and the White group while numerically different (AUC: 0.70 vs. 0.84, *p* = 0.74) did not reach statistical significance, with few events in the Black population. Due to limited data available about patient race, only subset of the cohort could be studied with limited number of events and the study is likely underpowered for such comparison.

### Association with outcomes and multivariable model

**Fig. 3:**
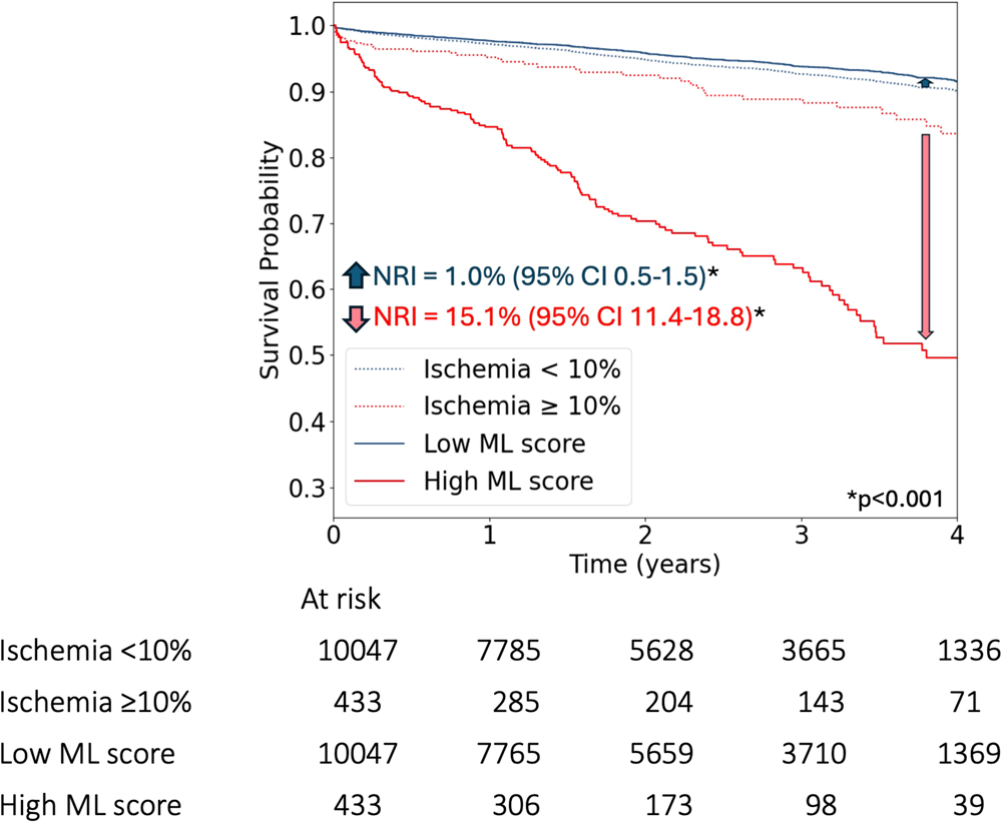
Kaplan-Meier (KM) curves stratified by total perfusion deficit (TPD). The KM Curves are matched to machine learning (ML) scores (All model). Ischemia was defined as TPD ≥ 10%. Abbreviations: CI – confidence interval, NRI – Net Reclassification Improvement.

Kaplan-Meier Curves stratified by TPD (ischemia <10% and ≥10%), and a matched proportion of patients with high and low AI scores (AI threshold at 0.17, high risk in 4.13%) are shown in Fig. 3. AI score led to an improved risk reclassification of patients who experienced mortality (15.1%, 95% CI 11.4–18.8, *p* < 0.001) and patients who did not experience mortality (1.0%, 95% CI 0.5–1.5, *p* < 0.001), with an overall net reclassification improvement of 16.1% (95% CI 12.4–19.8, *p* < 0.001). The stability of the AI threshold was assessed by inspecting the hazard ratios (HR) of the AI threshold in high-risk categorization across different subgroups in Supplementary Table 7. Notably, the mean adjusted HRs in all subgroups are above 4.

Supplementary Fig. 4 illustrates findings of multivariable analyses. After adjusting for age, sex (male), hypertension, dyslipidemia, diabetes mellitus, peripheral vascular disease, past myocardial infarction, and family history of CAD, patients with abnormal perfusion were at higher risk of death compared to patients with normal myocardial perfusion (adjusted HR 1.71, 95% CI 1.46–2.01, p < 0.001). Moreover, CAC > 400 (adjusted HR 2.11, 95% CI 1.67–2.65, *p* < 0.001) was associated with an increased risk of death.

### Structure specific risk evaluation

**Fig. 4:**
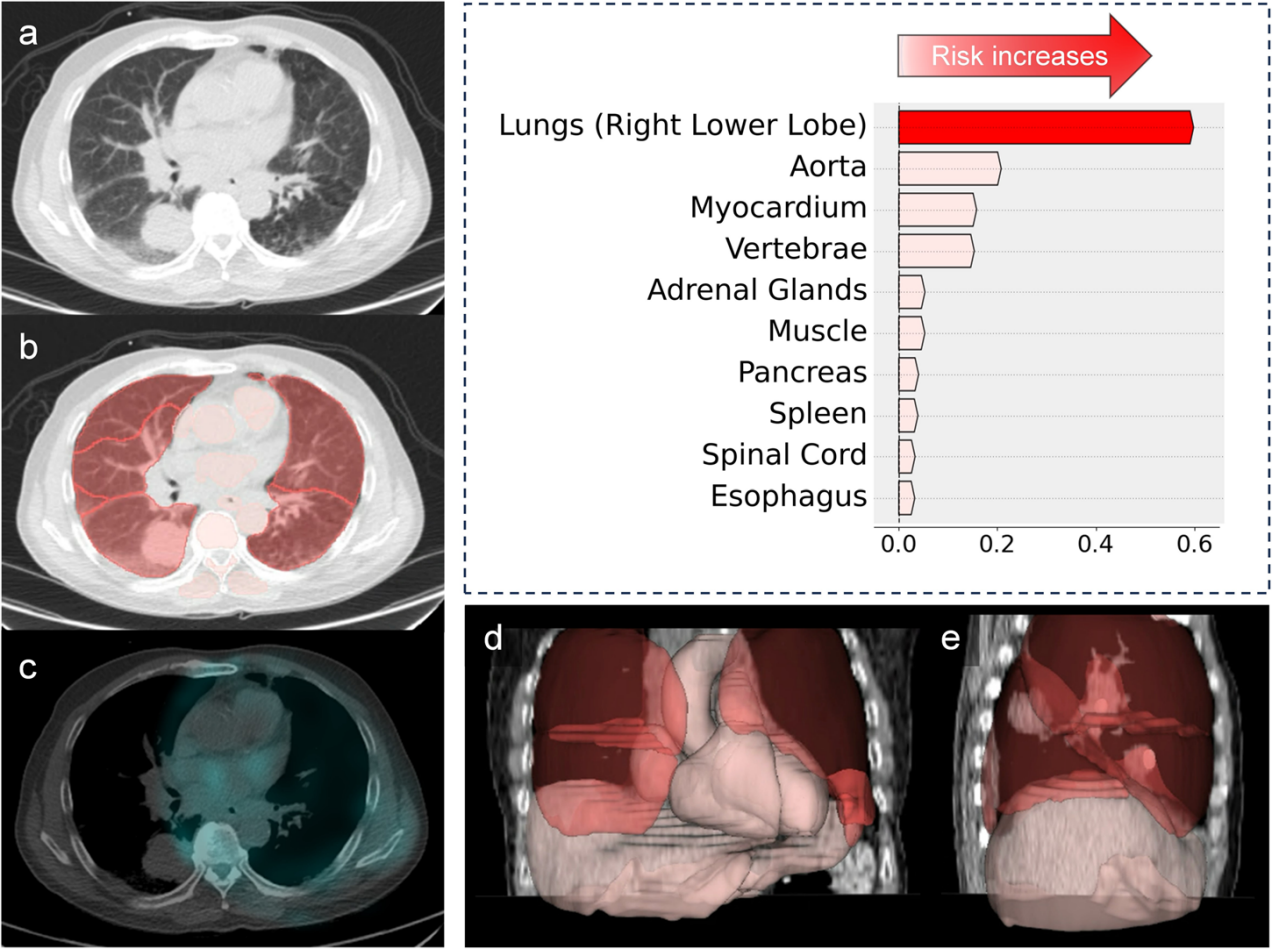
Example of a patient undergoing single-photon emission computed tomography/computed tomography (SPECT/CT) myocardial perfusion imaging with an extracardiac structure increasing the highest risk of all-cause mortality. Waterfall plot shows top 10 structures influencing mortality risk in the computed tomography attenuation correction (CTAC) model, highlighting Shapley Additive Explanations values (X-axis) and key structures. A 66-year-old male patient was classified to be at higher risk of death with the lungs (right lower lobe) contributing the most to the elevated risk (red arrow on the waterfall plot). **a** CTAC, axial view, with a corresponding deep learning structures segmentation (**b**) revealed a 39×39 mm solid mass with irregular margins in the right lower lobe. **c** CTAC with an overlayed SPECT scan showed no uptake of the radiotracer. **d, e** 3D reconstruction of all segmented and ranked structures. The patient had abnormal myocardial perfusion (total perfusion deficit of 7.65) and died 48 days after the exam.

Examples of patients classified to be at a higher risk of death (with extracardiac structures, notably the lungs and aorta, contributing the most to mortality) are shown in Fig. 4, Supplementary Figs. 5 and 6.

## Discussion

In this study, we have demonstrated the potential value of holistic anatomic, functional, and clinical evaluation of CTAC scans for improving all-cause mortality prediction in patients undergoing hybrid perfusion MPI. We developed a fully automated AI model incorporating multi-structure segmentation and radiomic feature extraction in parallel to deep learning-based CAC and EAT quantification. This model improves mortality prediction from multimodality myocardial perfusion, with a combined model improving upon any feature set (SPECT, CTAC, or clinical) in isolation. Moreover, it provides physicians with guidance regarding portions of CTAC scans which require further scrutiny to identify potentially important underlying conditions indicating potentially significant incidental findings, despite coronary artery disease being the primary indication for the examination. This fully automated workflow could be leveraged by physicians to unlock the full potential of hybrid SPECT/CT imaging.

Several studies have proven the role of AI in predicting mortality and cardiovascular events from cardiac imaging (Supplementary Table 1), only few of these studies were utilizing hybrid MPI^11,12^, and CTAC data^13,14^. None of the studies of the cardiovascular data considered comprehensively all organs in the field-of-view for the analysis. Moreover, only a limited number of CTAC findings, like CAC^13^ or EAT^10^ were included in these previous analyses. More recently we demonstrated that deep learning cardiac chamber volumes (from CTAC) provided incremental and complementary value to CAC and SPECT variables^15^. Ashrafinia et al. used radiomic features from SPECT MPI to predict CAC score derived from CT scans^16^, whereas Amini et al. applied a quantitative image analysis approach not only to diagnose CAD, but also for risk classification^17^. The proposed AI approach integrates simultaneous assessment of multiple structures on CTAC by leveraging strengths of deep learning and quantitative image analyses. Importantly, the model incorporating SPECT, CTAC, and clinical data had the highest prediction performance suggesting that AI-derived information encrypted in CTAC is complementary to traditional methods for analysis.

By integrating functional imaging (SPECT) with anatomic characteristics (CT), hybrid imaging has not only enhanced nuclear medicine by improving diagnostic accuracy^18^, but also provides an enormous amount of data contained in CTACs. This improvement was also observed in the performance of our model — the model including only perfusion and functional features performed significantly lower than the hybrid model (incorporating CTAC and SPECT data) or even the AI CTAC model alone. Moreover, the integration of clinical and imaging information improved the performance of the model in predicting the risk of death, which reflects the need for a holistic approach in patients’ diagnosis and radiology reporting^19^. While the 2024 ESC Guidelines for the Management of Chronic Coronary Syndromes recommend CAC scoring from CTACs to improve the detection of nonobstructive and obstructive CAD^20^, there is significantly more information in CTAC images beyond CAC that is not currently utilized. As demonstrated in this study, the highest feature importance score for predicting mortality was reported for the lungs. Although ischemic heart disease is the leading cause of mortality worldwide, the total number of lives lost due to respiratory diseases is still higher^21,22^. Incidental findings are frequently detected also on CTACs^6,7^, some of which may be clinically significant and require further diagnosis and treatment^23–25^. This underlines the need for a scrutinized evaluation of exams in patients undergoing diagnostic imaging for various reasons. For example, some respiratory diseases, like lung cancer and chronic obstructive pulmonary disease, share the same risk factors as CAD^22^ and early detection of potentially significant incidental findings might be lifesaving. AI-systems like the one proposed in our study could aid clinicians in these tasks.

This study has some limitations. It was a retrospective study with non-uniform CTAC acquisition protocols from multiple sites, however, this highlights the generalizability of the approach. Some organs (like the pancreas) were only partially visible or not visualized on all scans, whereas organs like kidneys and thyroid were excluded from the analysis because of their high missingness ( > 20%) across the cohort. For a more holistic approach and more accurate mortality prediction, organs with missingness <20% were included, however, this could influence model accuracy since, in some cases, fewer features were included. This large, multicenter registry does not include information on the reported cause of death, limiting our ability to evaluate the associations between specific extracardiac organ features and cause-specific mortality. Additionally, while SHAP and XGBoost are widely used for model explainability, their results can be subtly influenced by feature correlations and training data quality, highlighting the need for careful interpretation and oversight by clinicians. Another limitation of this study is the limited racial data, restricting subgroup analysis to Black and White individuals. Finally, radiological evaluation of CTACs was performed only with radiomic features, and no information regarding reported incidental findings is available in this cohort.

We demonstrate a significant, yet underappreciated, role of CTAC in risk stratification with MPI SPECT/CT. Fully automated AI integration of quantitative features from multiple organs derived from CTAC, perfusion and clinical data images significantly improves mortality risk stratification in patients undergoing SPECT/CT MPI as compared to MPI only.

## Material and methods

### Study population

In this retrospective study we utilized CTAC scans of patients who underwent SPECT/CT MPI from 4 sites (University of Calgary, Yale University, Columbia University, University of Ottawa Heart Institute) participating in the Registry of Fast Myocardial Perfusion Imaging with Next generation SPECT (REFINE SPECT)^26^. The cohort included consecutive patients at each center referred for SPECT imaging, with scans performed between 2009 and 2021. The study protocol complied with the Declaration of Helsinki and was approved by the institutional review boards (IRBs) at each participating institution, including the University of Calgary (Conjoint Health Research Ethics Board), Yale University School of Medicine (Human Research Protection Program, Institutional Review Boards), University of Ottawa Heart Institute (Ottawa Health Science Network Research Ethics Board), and Columbia University Irving Medical Center (Human Research Protection Program, Institutional Review Boards). The investigators ensured that the institutional ethics committee at each center evaluated and approved the study protocol before data collection and transfer. The overall study was approved by the institutional review board at Cedars-Sinai Medical Center (Office of Research Compliance and Quality Improvement). Sites either obtained written informed consent or waiver of consent for the use of the de-identified data. To the extent allowed by data sharing agreements and institutional review board protocols, the data and code from this manuscript will be shared upon written request. Baseline demographic and clinical characteristics were obtained from the REFINE SPECT registry^26^. CTAC image acquisition at each participating site is shown in Supplementary Table 8. The outcome was all-cause mortality (referred to subsequently simply as “mortality”), which was determined using the national death index for sites in the United States and administrative databases in Canada.

### Myocardial perfusion image analysis

Total perfusion deficit (TPD), end-diastolic stress shape index (ratio between the maximum left ventricular (LV) diameter in short axis and the length of the LV in end-diastole at stress), stress ejection fraction, and end-diastolic volume were quantified automatically from non-attenuation-corrected MPI scans at the core laboratory (Cedars-Sinai Medical Center, Los Angeles) with the use of dedicated software (Quantitative Perfusion SPECT [QPS] software, Cedars-Sinai Medical Center, Los Angeles)^27^. Normal myocardial perfusion was defined as stress TPD < 5%^28^, whereas moderate-to-severe ischemia was defined as TPD ≥ 10% of the myocardium^29^.

### Multi-structure deep learning feature extraction from CTAC

The study design is shown in Fig. 1. TotalSegmentator, a multi-structure segmentation deep learning (DL) model, was used to segment structures visible on CTAC^30^. Out of all segmented structures, we selected thirty-three structures with a frequency of >80% on all scans (Supplementary Fig. 7). The automatic extraction of imaging features for all selected structures was performed with PyRadiomics package (version 3.0.1)^31^. In per-organ analysis, we included eleven first-order and four 3D features which are clinically relevant and have straightforward clinical interpretation (Supplementary Tables 9-10).

One primary goal of this study was to create a simple, explainable model with high predictive power. We selected 15 radiomic features (11 first-order statistical and 4 3D shape-based) defined by PyRadiomics for their strong signal specificity and clinical relevance^32–35^. Grey-level features were excluded as they are deprecated in newer radiomics versions^36^. Further, we conducted a comparison between the performance of the models created with all calculated 32 radiomic features and the subset of clinically interpretable 15 radiomic features (for the names of these selected features please see Supplementary Table 9). There were no statistically significant differences in performance between the models using all 32 radiomic features and those using 15 features for the All and AI CTAC models (*p* = 0.09 and *p* = 0.40, respectively, Supplementary Table 10). Additionally, for the AI hybrid model, the 15-feature subset performed significantly better than the full 32-feature set (see Supplementary Table 10 for AUCs, confidence intervals, and *p*-values). This supports our decision to use the clinically interpretable 15-feature subset, as it simplifies the model without compromising performance and, in some cases, enhances it.

### Automated coronary artery calcium scoring

Our formerly validated deep learning model was used for CAC segmentation and scoring^37,38^. To segment heart mask and CAC on CTAC images, two convolutional long short-term memory (convLSTM) networks were tested externally on data (10,480 CTAC scans) from 4 different sites. To automatically obtain CAC scores from the deep learning segmentation, established methods were used^39^.

### Automated epicardial adipose tissue scoring

A previously developed deep learning model was used to estimate EAT volume and density (−190 and −30 Hounsfield units [HU]) from CTAC scans^10^. For EAT model training and validation purposes, we used 500 CTAC scans from one site (Yale University). Patients who were used for EAT model training and validation were not included in this analysis.

### Classification models

Extreme Gradient Boosting (XGBoost) models (version 1.7.3), a currently leading machine learning method, were used for mortality classification^33^. These models generate all-cause mortality risk scores by applying 10-fold cross-validation regimen across the entire dataset. Within each fold, 90% of the data was first set aside for model training and validation. This 90% was further divided, with 80% used for training and 20% for validation. The remaining 10% of the data in each fold was used for testing and kept separate from training and validation to ensure each patient was tested exactly once across all folds. 10 separate models were built, and each was tested independently. Testing results were concatenated from all models for the overall performance evaluation. Hyper-parameter tuning to optimize the model parameters was conducted during training and validation, separately in each fold using the grid-search method.

Key benefits of employing 10-fold cross-validation include: 1) reducing variability of prediction errors for more accurate evaluation^40^; 2) maximizing data utilization while minimizing overfitting and cross-contamination of information among data splits^41^; 3) ensuring each data point contributes to the test set exactly once, providing independent and non-overlapping predictions for robust performance evaluation^42^; 4) meeting the DeLong test requirements for valid AUC comparisons by using independent predictions^43^.

### Models

Five models were used for the mortality endpoint: 1 – model incorporating DL-EAT (**EAT**), 2 – model combining quantitative CTAC image analysis of all segmented structures [radiomics], DL-EAT and DL-CAC **(AI CTAC**), 3 – model incorporating stress ejection fraction, stress end-diastolic volume, stress shape index end-diastolic, stress TPD, and other SPECT imaging features (in total 22 features) [see Supplementary Table 11] **(AI SPECT)**, 4 – model incorporating all variables included in the AI CTAC model as well features included in the AI SPECT model **(AI hybrid)**, 5 – model combining CTAC, MPI and clinical data (**All)**, whereas **Coronary calcium** (DL-CAC score) and **Perfusion** (utilizing stress TPD) were univariate comparisons.

Clinical data include patient demographics such as age, sex, body mass index (BMI). Also included is past medical history: hypertension, diabetes, dyslipidemia, prior CAD (prior myocardial infarction, percutaneous coronary intervention [PCI], and coronary artery bypass graft [CABG]). Further, the clinical data encompass variables from stress test such as the type of test, peak stress heart rate, peak stress blood pressure, and ECG response to stress.

### Model explainability

The predictive power of variables included in model training was evaluated using XGBoost feature importance, which quantifies the increase in accuracy resulting from the addition of each feature. SHapley Additive explanations (SHAP), a game-theoretic feature importance method, was used to explain how structures contributed to the overall risk in model inference for individual patients^44^.

### Thresholds for comparisons of machine learning

Patients were classified into low or high-risk groups based on AI-derived all-cause mortality risk score. This classification was achieved by setting a threshold that aligns with the proportion of patients identified by the established clinical criteria for ischemia ( ≥ 10%)^45,46^.

### Statistical analysis

Continuous variables with a normal distribution are presented as mean ± standard deviation (SD) and not normally distributed variables as medians with interquartile range (IQR) [IQ1-IQ3]. Categorical variables are expressed as count and relative frequencies (percentages). Differences between categorical variables were compared by the Pearson’s χ2 test whereas continuous variables were compared by Wilcoxon Mann-Whitney test, as appropriate. The performance of the models was evaluated using receiver-operating characteristics analysis, and area under the receiver-operating characteristic (AUC) analysis values were compared with the DeLong test^47^. Kaplan-Meier survival curve, alongside univariate Cox proportional hazard models, were employed to evaluate the association with mortality. Log-rank test was used to ascertain the statistical significance. The improvement in model predictions was measured using the time-dependent net reclassification improvement score at 2 years^48^. Confidence intervals were calculated by the percentile bootstrap method. A two-tailed *p*-value of <0.05 was considered statistically significant. All statistical analyses were performed with Pandas (version 2.1.1) and Numpy (version 1.24.3), Scipy (version 1.11.4), Lifelines (version 0.28.0) and Scikit-learn (version 1.3.0) in Python 3.11.5 (Python Software Foundation, Wilmington, DE, USA), as well as “nricens” package (version 1.6) in R version 4.3.2 (R Foundation for Statistical Computing, Vienna, Austria).

## Supplementary information

Supplementary Material

## Data Availability

To the extent allowed by data sharing agreements and IRB protocols, the data from this manuscript will be shared upon written request.

## Code availability

The TotalSegmentator code is publicly available^15^. cLSTM code is publicly available under a Creative Commons BY-NC license at 10.5281/zenodo.10632288. PyRadiomics code is available at https://pyradiomics.readthedocs.io/en/latest/. The XGBoost Python implementation is available at https://xgboost.readthedocs.io/en/stable/python/. The analysis code and the model used in this work will be made available on GitHub: https://github.com/qimagingAI/HolisticAIforSPECT-CT.
