# Supplementary material for: Holistic AI analysis of hybrid cardiac perfusion images for mortality prediction
Source: NPJ Digit Med. 2025 Mar 13;8:158. doi: 10.1038/s41746-025-01526-0 (PMC11906890; doi:10.1038/s41746-025-01526-0)
Supplement: Supplementary file 1 — Supplementary Material [file 41746_2025_1526_MOESM1_ESM.docx]

**SUPPLEMENTARY MATERIAL**

Supplementary Figure 1 Page 2

Supplementary Figure 2 Page 3

Supplementary Figure 3 Page 4

Supplementary Figure 4 Page 5-6

Supplementary Figure 5 Page 7

Supplementary Figure 6 Page 8

Supplementary Figure 7 Page 9

Supplementary Table 1 Page 10-11

Supplementary Table 2 Page 12-13

Supplementary Table 3 Page 14

Supplementary Table 4 Page 15

Supplementary Table 5 Page 16

Supplementary Table 6 Page 17

Supplementary Table 7 Page 18

Supplementary Table 8 Page 19

Supplementary Table 9 Page 20

Supplementary Table 10 Page 21

Supplementary Table 11 Page 22

**
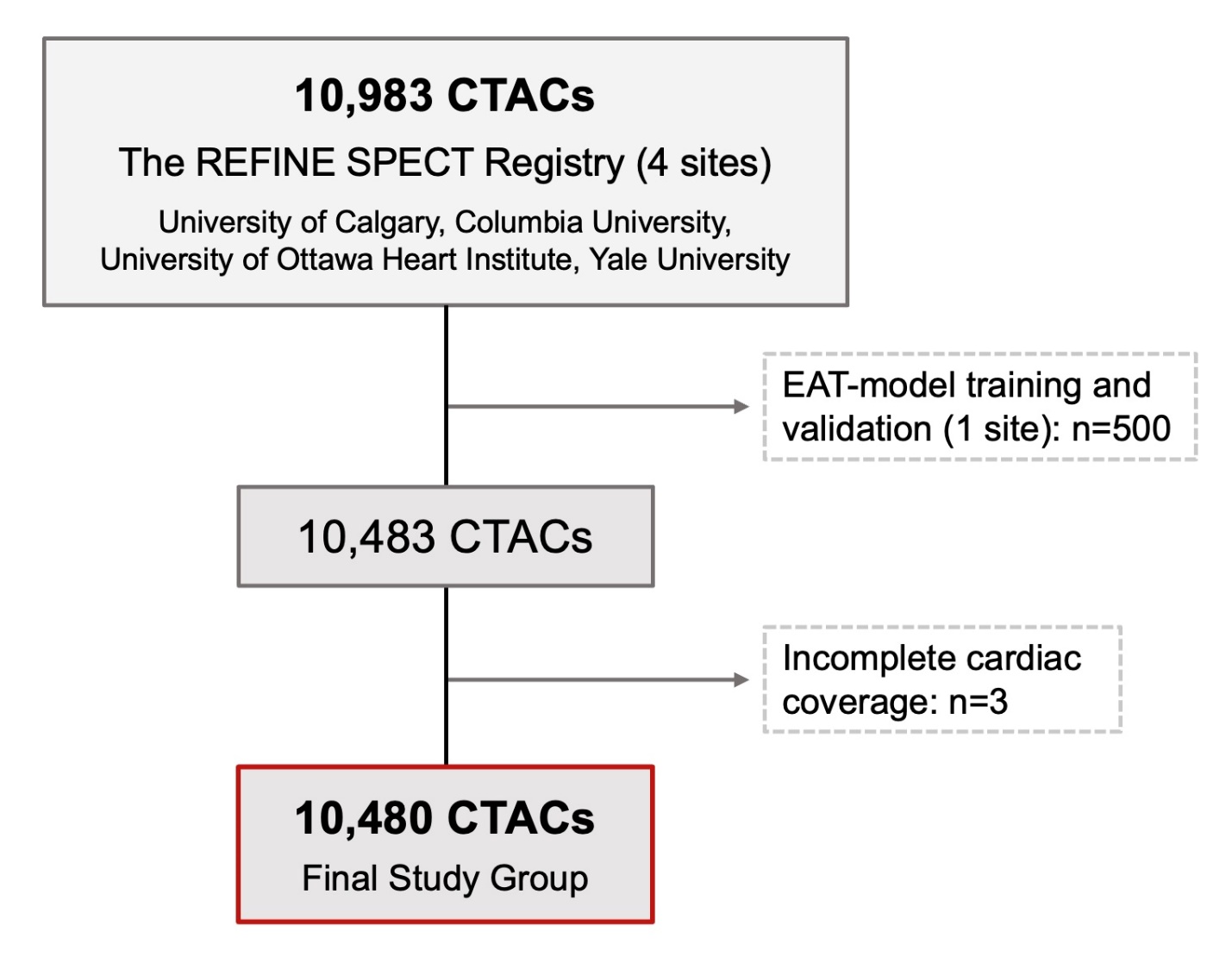
**

**Supplementary Figure 1. Flowchart.** A graphical illustration depicts the study cohorts analyzed and the inclusion criteria applied for the analysis. Abbreviations: CTAC – computed tomography attenuation correction; EAT – epicardial adipose tissue; SPECT – single-photon emission computed tomography.

**
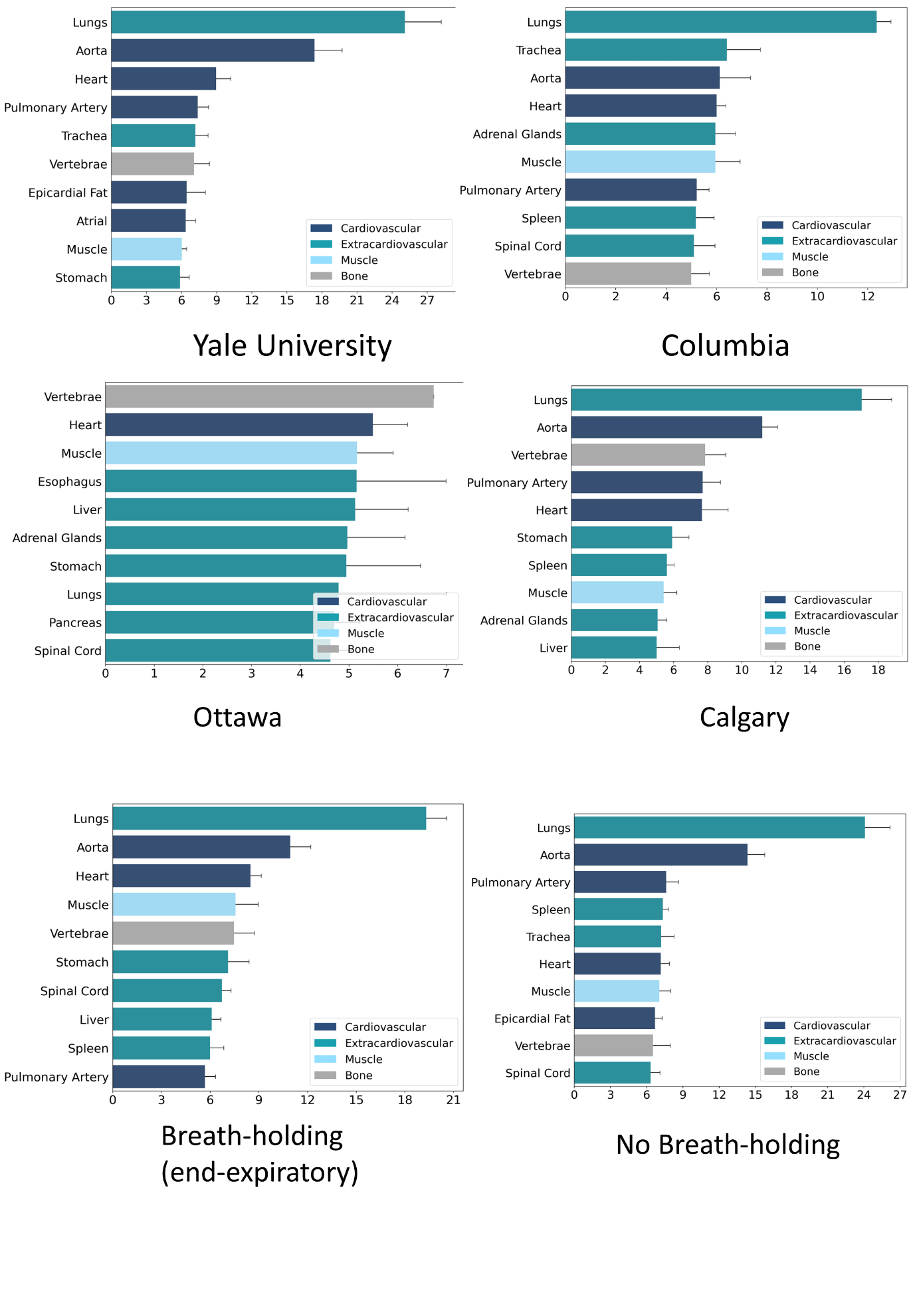
**

**Supplementary Figure 2**. **Feature importance scores for all-cause mortality.** The scores were generated by XGBoost by different sites and acquisition protocols.


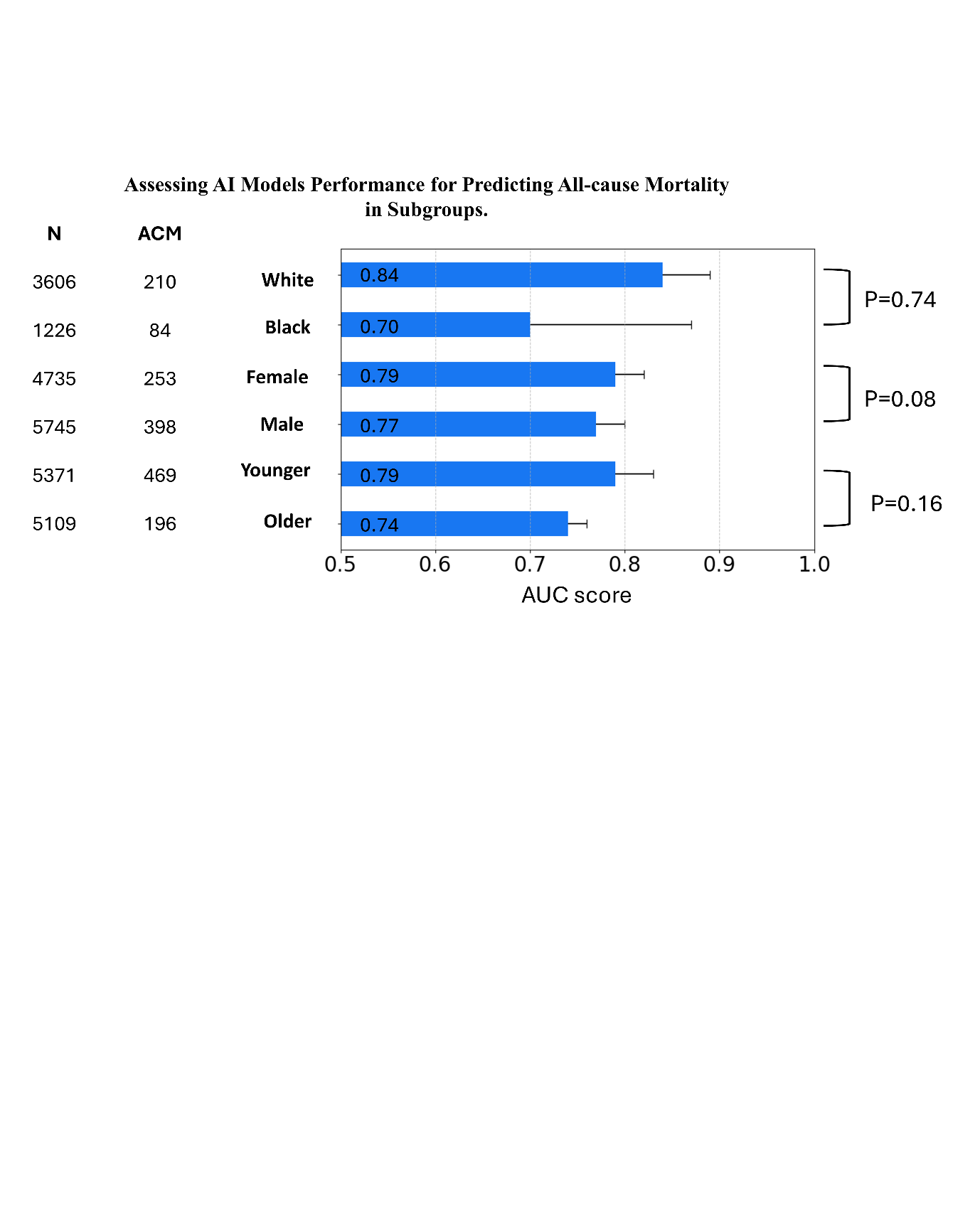


**Supplementary Figure 3.** **Assessing AI models performance for predicting all-cause mortality in subgroups.** The following groups were used: white race, black race, female, male, older (≥65-year-old), and younger (<65-year-old). Due to limited data for other races, the race-based subgroup analysis was restricted to black and white individuals. The number on the bars are the mean AUC scores for each group. Unpaired Delong test (pROC library in R, version 1.18.5) was used for p-values. Abbreviations: ACM – All Cause Mortality; AI – Artificial Intelligence; AUC – Area Under the Receiver Operating Characteristic Curve.

**
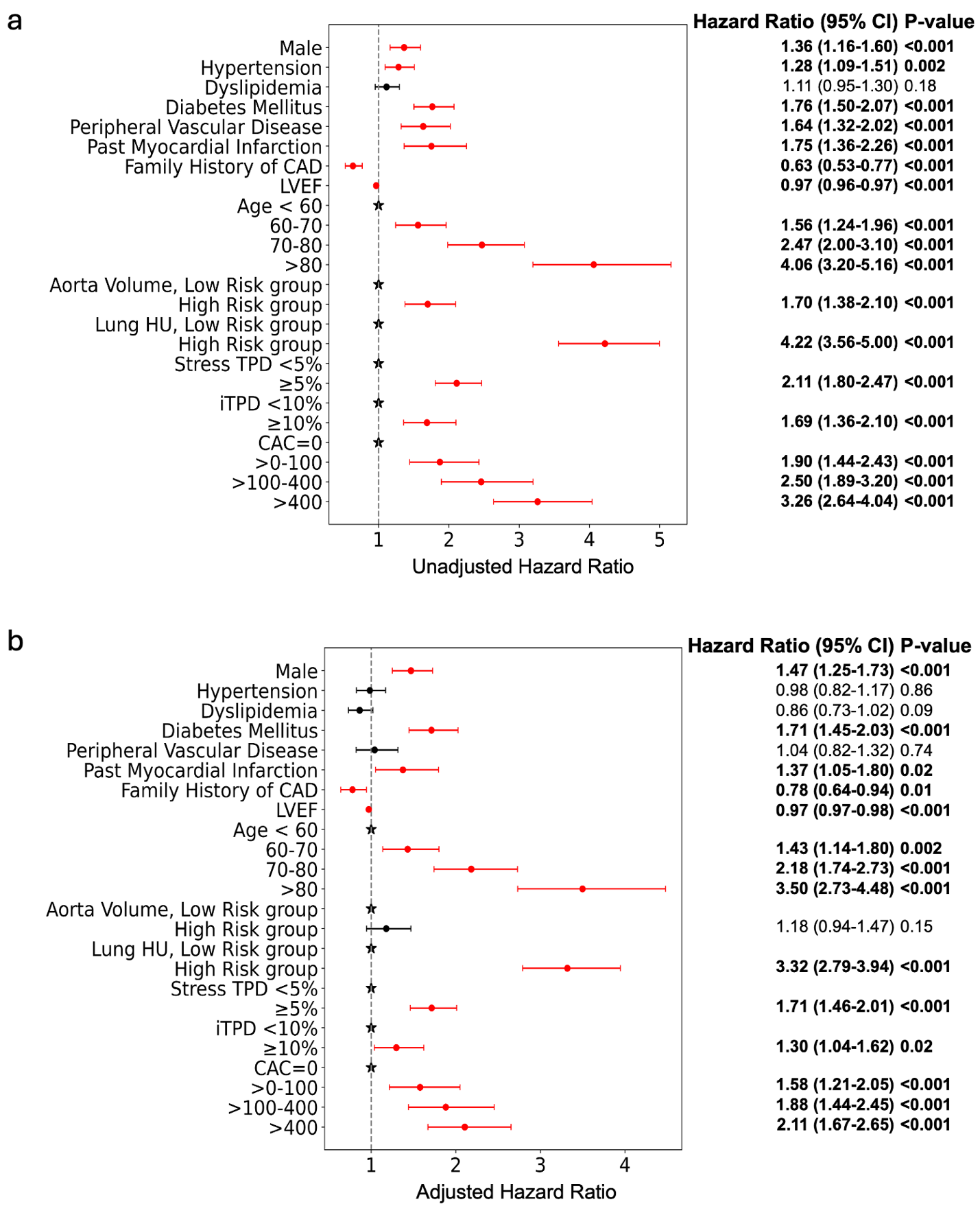
**

**Supplementary Figure 4. Forest plot of the unadjusted (a) and adjusted (b) hazard ratio and 95% confidence interval (CI) for death.** Adjusted factors: age, sex (male), hypertension, dyslipidemia, diabetes mellitus, peripheral vascular disease, past myocardial infarction, family history of coronary artery disease (CAD). Abbreviations: CAC – coronary artery calcium, HU – Hounsfield units, iTPD – ischemic total perfusion deficit, LVEF – left ventricular ejection fraction, TPD – total perfusion deficit.

**
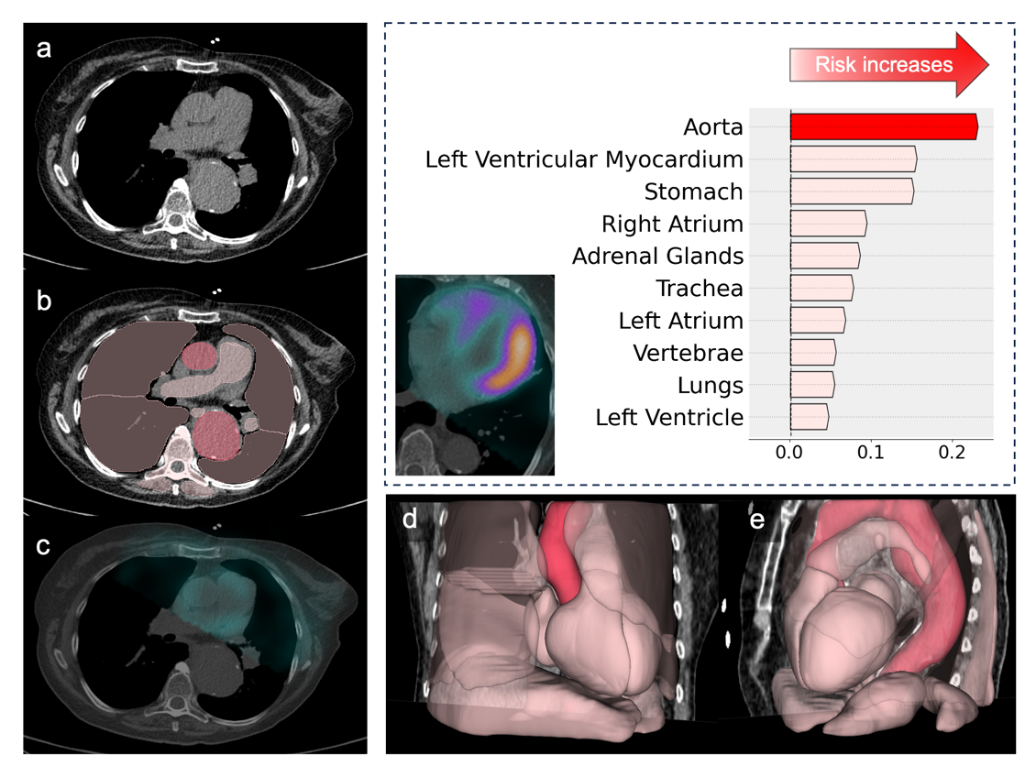
**

**Supplementary Figure 5.** **Example of a patient undergoing single-photon emission computed tomography/computed tomography (SPECT/CT) myocardial perfusion imaging with an extracardiac structure increasing the highest risk of all-cause mortality.** A 76-year-old male patient was identified to be at elevated risk of mortality. The risk of death was increased the most by the aorta (red arrow on the waterfall plot). Waterfall plot shows top 10 structures influencing mortality risk in the computed tomography attenuation correction (CTAC) model, highlighting Shapley Additive Explanations values (X-axis) and key structures. **a.** CTAC, axial view, with a corresponding deep learning (DL) structures segmentation (**b**) showed a descending aortic aneurysm (cross-sectional diameters of the descending aorta 53x51 mm at the level of the pulmonary artery bifurcation). **c.** CTAC with an overlayed SPECT scan, coronal view. **d-e.** 3D reconstruction of all segmented and ranked structures. The patient had abnormal myocardial perfusion (total perfusion deficit of 32.1) and died 209 days after the exam.

**
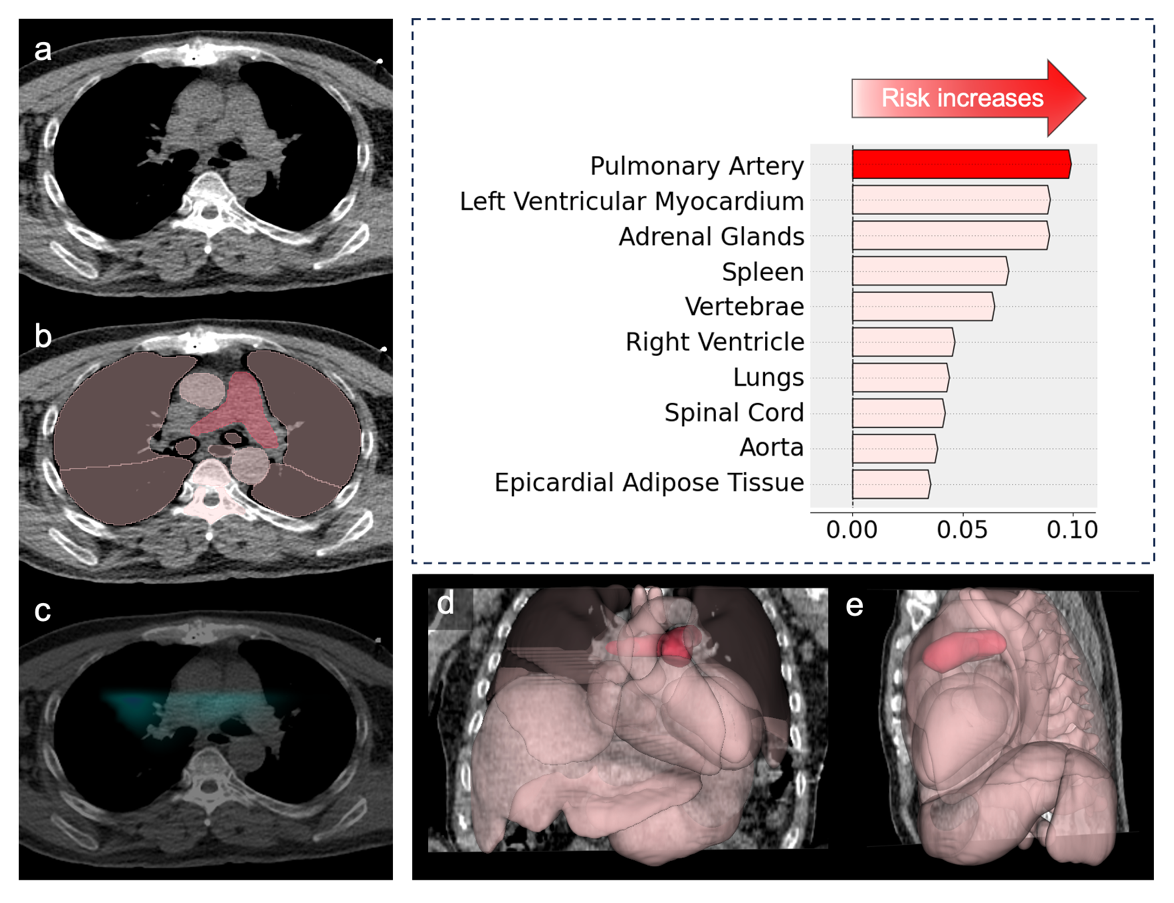
**

**Supplementary Figure 6. Example of a patient undergoing single-photon emission computed tomography/computed tomography (SPECT/CT) myocardial perfusion imaging with an extracardiac structure increasing the highest risk of all-cause mortality.** The dilated pulmonary artery was contributing the most to the elevated risk (red arrow on the waterfall plot) of death in a 54-year-old male patient. Waterfall plot shows top 10 structures influencing mortality risk in the computed tomography attenuation correction (CTAC) model, highlighting Shapley Additive Explanations values (X-axis) and key structures. **a.** CTAC with a corresponding deep learning structures segmentation (**b**) showed a dilatated pulmonary artery (the pulmonary artery diameter on transaxial image - 32 mm). **c.** CTAC with an overlayed SPECT scan showed no uptake of the radiotracer. **d-e**. 3D reconstruction of all segmented and ranked structures. The patient had abnormal myocardial perfusion (total perfusion deficit of 9.67) and died 5.4 years after the exam.


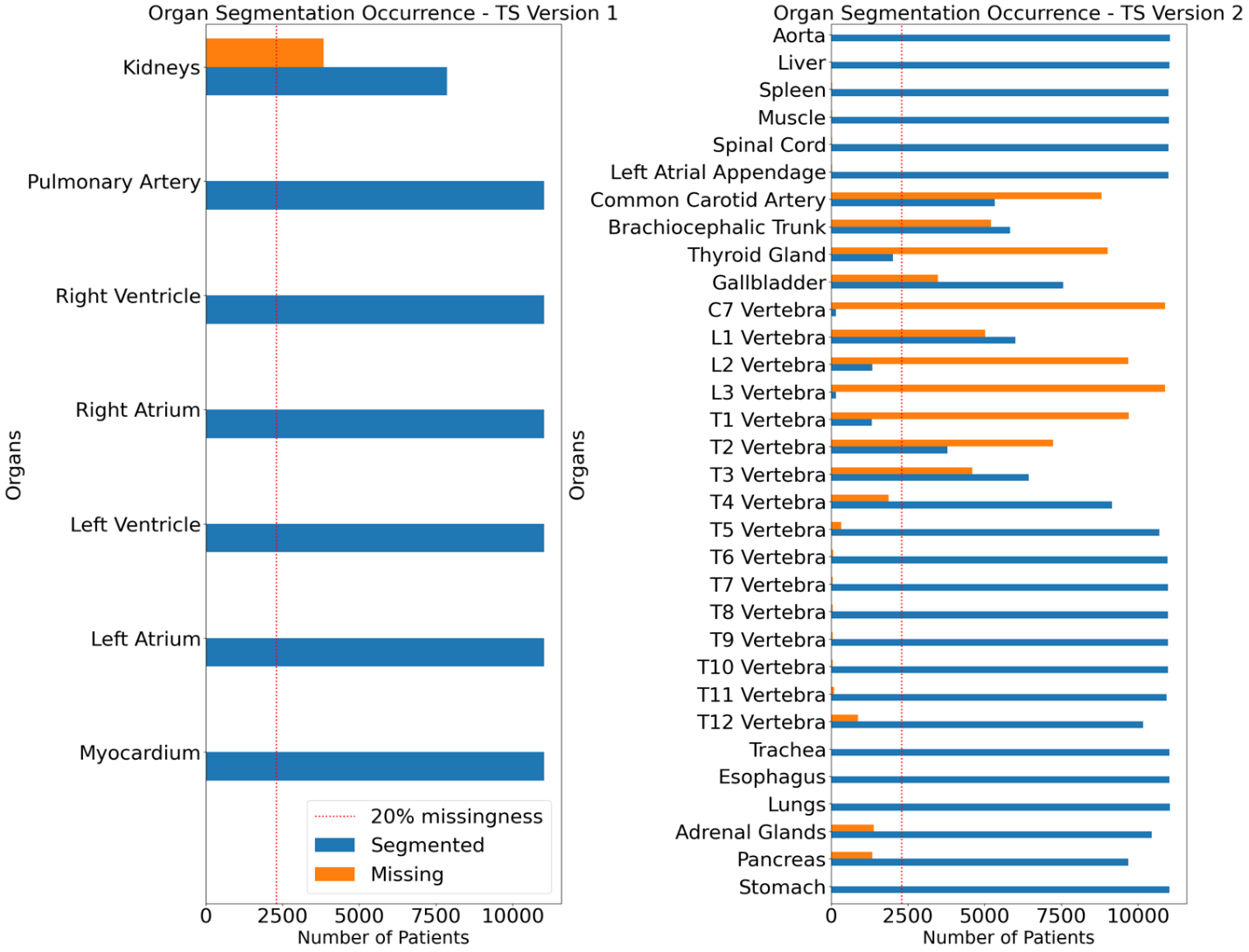


**Supplementary Figure 7.** **Histogram of successfully segmented structures.** The visible structures on computed tomography attenuation correction (CTAC) scans were segmented with a use of deep-learning models. Structures present in >80% of CTAC were included. Abbreviations: TS – TotalSegmentator.

**Supplementary Table 1. Related works on artificial intelligence-based all-cause mortality (ACM) or major adverse coronary events (MACE) prediction from cardiac imaging.**

|  | Title | Type of Exam | Prediction |
| --- | --- | --- | --- |
| 1 | Miller RJH et al. Deep Learning Coronary Artery Calcium Scores from SPECT/CT Attenuation Maps Improve Prediction of Major Adverse Cardiac Events. J Nucl Med. 2023 Apr;64(4):652-658. | SPECT/CT | MACE |
| 2 | Alahdab F et al. Patient-level explainable machine learning to predict major adverse cardiovascular events from SPECT MPI and CCTA imaging. PLoS One 18, e0291451 (2023). | SPECT/CT | MACE |
| 3. | Cheng DC et al. Prediction of All-Cause Mortality Based on Stress/Rest Myocardial Perfusion Imaging (MPI) Using Deep Learning: A Comparison between Image and Frequency Spectra as Input. J Pers Med. 2022 Jul 5;12(7):1105. | SPECT | ACM |
| 4 | Singh A et al. Deep Learning for Explainable Estimation of Mortality Risk From Myocardial Positron Emission Tomography Images. Circ Cardiovasc Imaging. 2022 Sep;15(9):e014526. | PET/CT | ACM |
| 5 | Singh A et al. Direct Risk Assessment From Myocardial Perfusion Imaging Using Explainable Deep Learning. JACC Cardiovasc Imaging. 2023 Feb;16(2):209-220. | PET | ACM, MACE |
| 6 | Pieszko K et al. Time and event-specific deep learning for personalized risk assessment after cardiac perfusion imaging. NPJ Digit Med. 2023 May 1;6(1):78. | PET | ACM |
| 7 | Juarez-Orozco LE et al. Hybridizing machine learning in survival analysis of cardiac PET/CT imaging. J Nucl Cardiol. 2023 Dec;30(6):2750-2759. | PET/CT | ACM |
| 8 | Tremamunno G et al. Artificial Intelligence Improves Prediction of Major Adverse Cardiovascular Events in Patients Undergoing Transcatheter Aortic Valve Replacement Planning CT. Acad Radiol. 2024 Oct 9:S1076-6332(24)00695-0. | CCTA | MACE |
| 9 | Motwani M et al. Machine learning for prediction of all-cause mortality in patients with suspected coronary artery disease: a 5-year multicentre prospective registry analysis. Eur Heart J. 2017 Feb 14;38(7):500-507. | CCTA | ACM |
| 10 | van Rosendael AR et al. Maximization of the usage of coronary CTA derived plaque information using a machine learning based algorithm to improve risk stratification; insights from the CONFIRM registry. J Cardiovasc Comput Tomogr. 2018 May-Jun;12(3):204-209. | CCTA | MACE |
| 11 | O'Driscoll JM et al. Artificial intelligence calculated global longitudinal strain and left ventricular ejection fraction predicts cardiac events and all-cause mortality in patients with chest pain. Echocardiography. 2023 Dec;40(12):1356-1364. | TTE | ACM, MACE |
| 12 | Valsaraj A et al. Development and validation of echocardiography-based machine-learning models to predict mortality. EBioMedicine. 2023 Apr;90:104479. | TTE | ACM |
| 13 | Lancaster MC et al. Phenotypic Clustering of Left Ventricular Diastolic Function Parameters: Patterns and Prognostic Relevance. JACC Cardiovasc Imaging. 2019 Jul;12(7 Pt 1):1149-1161. | TTE | ACM,  MACE |
| 14. | Alabed S et al. Validation of Artificial Intelligence Cardiac MRI Measurements: Relationship to Heart Catheterization and Mortality Prediction. Radiology. 2022 Oct;305(1):68-79. | MRI | ACM |
| 15 | Ishikita A et al. Machine Learning for Prediction of Adverse Cardiovascular Events in Adults With Repaired Tetralogy of Fallot Using Clinical and Cardiovascular Magnetic Resonance Imaging Variables. Circ Cardiovasc Imaging. 2023 Jun;16(6):e015205. | MRI | MACE |
| 16. | Corianò M et al. Deep learning-based prediction of major arrhythmic events in dilated cardiomyopathy: A proof of concept study. PLoS One. 2024 Feb 29;19(2):e0297793. | MRI | MAE (including sudden cardiac death) |

CCTA – coronary computed tomography angiography, CT – computed tomography, MAE – major arrhythmic events, MPI – myocardial perfusion imaging, MRI – magnetic resonance imaging, PET -positron emission tomography, SPECT - single-photon emission computed tomography, TTE – transthoracic echocardiography

**Supplementary Table 2.** Baseline characteristics for all participants stratified by TPD

|  | All Participants | Abnormal Perfusion | Normal Perfusion | P-value |
| --- | --- | --- | --- | --- |
| N (%) | 10,480 | 3,151 | 7,329 |  |
| Age [years] | 65 (57, 73) | 67 (58, 75) | 64 (56, 73) | <0.001 |
| Male | 5,745 (55) | 1,842 (58.5) | 3,903 (53.3) | <0.001 |
| BMI [kg/m^2^] | 29 (25, 33) | 29 (25, 34) | 28 (25, 33) | <0.001 |
| Hypertension | 6,175 (58.9) | 1,965 (62.4) | 4,210 (57.4) | <0.001 |
| Diabetes mellitus | 2,681 (25.6) | 1,009 (32.0) | 1,675 (22.9) | <0.001 |
| Dyslipidemia | 5,085 (48.5) | 1,634 (51.9) | 3,451 (47.1) | <0.001 |
| Smoking | 1,987 (19.0) | 560 (17.8) | 1,427 (19.5) | 0.046 |
| Family history of CAD | 2,771 (26.4) | 796 (25.3) | 1,975 (26.9) | 0.077 |
| Prior CAD |  |  |  |  |
| Prior Myocardial Infarction | 750 (7.2) | 394 (12.5) | 356 (4.9) | <0.001 |
| Past PCI | 1,508 (14) | 737 (23.4) | 771 (10.5) | <0.001 |
| Past CABG | 636 (6.1) | 388 (12.3) | 248 (3.4) | <0.001 |
| Mortality | 651 (6.2) | 306 (9.7) | 345 (4.7) | <0.001 |
| CT Quantitative Image Analysis Parameters | | | | |
| DL-CAC score | 56 (0, 709) | 239 (0, 1404) | 29 (0, 424) | <0.001 |
| DL-EAT volume [mL] | 130 (90, 183) | 133 (93, 191) | 129 (89, 181) | 0.001 |
| DL-EAT density [HU] | -65 (-70, -61) | -65 (-70, -61) | -65 (-70, -61) | 0.019 |
| MPI Acquisition Parameters |  |  |  |  |
| Stress Test Type |  |  |  | <0.001 |
| Exercise | 4,732 (45.2) | 1,177 (37.4) | 3,555 (48.5) |  |
| Pharmacological | 5,748 (54.8) | 1,974 (62.6) | 3,774 (51.5) |  |
| Peak Stress Heart Rate | 112 (89, 146) | 102 (84, 134) | 118 (92, 150) | <0.001 |
| Peak Stress Systolic Blood Pressure | 148 (128, 170) | 140 (121, 164) | 150 (130, 172) | <0.001 |
| Peak Stress Diastolic Blood Pressure | 80 (70, 86) | 78 (70, 84) | 80 (70, 88) | <0.001 |
| ECG Response to Stress |  |  |  | <0.001 |
| Negative | 8,010 (76.4) | 2,266 (71.9) | 5,744 (78.4) |  |
| Positive | 1,167 (11.1) | 415 (13.2) | 752 (10.3) |  |
| Equivocal | 455 (4.3) | 111 (3.5) | 344 (4.7) |  |
| Nondiagnostic | 824 (7.9) | 352 (11.2) | 472 (6.4) |  |
| Borderline | 10 (<0.1) | 3 (<0.1) | 7 (<0.1) |  |
| MPI Quantitative Image Analysis Parameters | | | |  |
| Stress Ejection Fraction | 64 (55, 72) | 57 (46, 67) | 66 (59, 73) | <0.001 |
| Stress End Diastolic Volume | 84 (64, 111) | 97 (71, 132) | 80 (62, 103) | <0.001 |
| Stress Shape Index End Diastolic | 0.58 (0.54, 0.62) | 0.60 (0.55, 0.65) | 0.57 (0.53, 0.61) | <0.001 |
| Stress Total Perfusion Deficit | 2.6 (0.9, 6.0) | 8.9 (6.5, 14.2) | 1.5 (0.4, 2.8) | <0.001 |

Values are presented as N (%) or median (IQ1, IQ3); Normal perfusion – TPD <5%

BMI – body mass index; CABG – coronary artery bypass graft; CAC – coronary artery calcium; CAD – coronary artery disease; CT – computed tomography; DL – deep learning; EAT– epicardial adipose tissue; ECG–electrocardiogram; HU – Hounsfield units; MACE – major adverse cardiovascular events; MPI – myocardial perfusion imaging; N – number of patients; PCI – percutaneous coronary intervention; TPD – total perfusion deficit

**Supplementary Table 3.** Area under the receiver-operating characteristic curve (AUC) with 95% confidence interval (CI) for all artificial intelligence models and coronary calcium and perfusion (stress total perfusion deficit) in all patients.

| Model | AUC | 95% CI | P-value |
| --- | --- | --- | --- |
| All | 0.80 | 0.74-0.87 | Reference |
| AI hybrid | 0.79 | 0.72-0.86 | <0.001 |
| AI CTAC | 0.78 | 0.71-0.85 | <0.001 |
| AI SPECT | 0.65 | 0.58-0.72 | <0.001 |
| Coronary calcium | 0.64 | 0.57-0.71 | <0.001 |
| Perfusion | 0.62 | 0.55-0.70 | <0.001 |
| EAT | 0.56 | 0.49-0.63 | <0.001 |

The models are described in the Methods section.

AI – artificial intelligence; CTAC – computed tomography attenuation correction; EAT – epicardial adipose tissue; SPECT – single photon emission computed tomography.

**Supplementary Table 4.** Area under the receiver-operating characteristic curve (AUC) with 95% confidence interval (CI) for all artificial intelligence models, coronary calcium and perfusion (stress total perfusion deficit) in patients with normal myocardial perfusion.

| Model | AUC | 95% CI | P-value |
| --- | --- | --- | --- |
| All | 0.78 | 0.70-0.86 | Reference |
| AI hybrid | 0.76 | 0.68-0.84 | 0.004 |
| AI CTAC | 0.76 | 0.67-0.84 | 0.001 |
| Coronary calcium | 0.63 | 0.54-0.72 | <0.001 |
| EAT | 0.54 | 0.45-0.64 | <0.001 |
| Perfusion | 0.53 | 0.43-0.63 | <0.001 |

The models are described in the Methods section.

AI – artificial intelligence; CTAC – computed tomography attenuation correction; EAT – epicardial adipose tissue.

**Supplementary Table 5.** Area under the receiver-operating characteristic curve (AUC) with 95% confidence interval (CI) for all artificial intelligence models, coronary calcium and perfusion (stress total perfusion deficit) in patients with no coronary calcium.

| Model | AUC | 95% CI | P-value |
| --- | --- | --- | --- |
| All | 0.78 | 0.63-0.94 | Reference |
| AI hybrid | 0.75 | 0.60-0.92 | <0.001 |
| AI CTAC | 0.71 | 0.55-0.88 | <0.001 |
| EAT | 0.59 | 0.42-0.76 | <0.001 |
| Perfusion | 0.59 | 0.41-0.76 | <0.001 |
| Coronary calcium | 0.50 | 0.33-0.67 | <0.001 |

The models are described in the Methods section.

AI – artificial intelligence; CTAC – computed tomography attenuation correction; EAT – epicardial adipose tissue.

**Supplementary Table 6**. Assessing AI models performance for predicting all-cause mortality stratified by sites, breath-holding, and slice thickness acquisition protocols. The area under the receiver operating characteristic curve was presented with mean and 95% confidence intervals.

|  | N | ACM (%) | AUC (All model) | P value^†^ |
| --- | --- | --- | --- | --- |
| Yale | 4,266 | 233 (5.5%) | 0.82 [0.79, 0.85] | Reference |
| Calgary | 2,912 | 261 (9.0%) | 0.80 [0.77, 0.83] | 0.20 |
| Columbia | 1,876 | 127 (6.8%) | 0.72 [0.68, 0.76] | <0.001 |
| Ottawa | 1,426 | 30 (2.1%) | 0.69 [0.59, 0.79] | <0.001 |
| Breath-holding |  |  |  |  |
| Yes (end-expiratory) | 4,338 | 291 (6.7%) | 0.82 [0.79, 0.85] | Reference |
| No | 6,142 | 360 (5.9%) | 0.79 [0.77, 0.81] | 0.72 |
| Slice thickness (mm) |  |  |  |  |
| 5.0 | 4,338 | 291 (6.7%) | 0.82 [0.79, 0.85] | Reference |
| ≤3.0 | 6,142 | 360 (5.9%) | 0.79 [0.77, 0.81] | 0.72 |
| Tube current (mA) |  |  |  |  |
| 60/150* | 4,266 | 233 (5.5%) | 0.82 [0.79, 0.85] | Reference |
| ≤30 | 6,214 | 418 (6.7%) | 0.79 [0.77, 0.81] | 0.17 |

ACM – All Cause Mortality; AI – Artificial Intelligence; AUC – Area Under the Receiver Operating Characteristic Curve.

*Acquisition parameters adjusted for patients with body mass index ≥ 40 kg/m^2^.

† Unpaired Delong test (pROC library in R, version 1.18.5) was used for p values.

**Supplementary Table 7**. Assessing the stability of adjusted hazard ratios (HR) for the AI threshold in high-risk categorization across different groups.

| Groups | HR of AI high-risk* | p value | N | ACM |
| --- | --- | --- | --- | --- |
| Male | 4.16 [2.92, 5.91] | p <0.001 | 5,745 | 398 |
| Female | 4.62 [2.81, 7.61] | p <0.001 | 4,735 | 253 |
| Older (≥65 years) | 4.53 [3.30, 6.21] | p <0.001 | 5,109 | 196 |
| Younger (<65 years) | 4.93 [2.57, 9.45] | p <0.001 | 5,371 | 469 |
| White | 4.41 [3.11, 6.25] | p <0.001 | 3,606 | 210 |
| Black | 4.38 [2.18, 8.81] | p <0.001 | 1,226 | 84 |

* Hazard ratios (HR) were written as mean [95% confidence interval]; HR were adjusted by age, gender, race, hypertension, dyslipidemia, diabetes mellitus, peripheral vascular disease, smoking, past myocardial infarction, family history, and stress test type. Note that the relative covariate was excluded from adjustment; for instance, age was not included as an adjustment factor when analyzing older or younger groups.

ACM – All Cause Mortality; AI – Artificial Intelligence.

**Supplementary Table 8**. Computed tomography attenuation correction image acquisition parameters

| Site | University of Calgary | Yale University | Columbia | Ottawa |
| --- | --- | --- | --- | --- |
| Scanner | GE Discovery NM/CT 570c | GE Discovery NM/CT 570c | Phillips Precedence 16P | Siemens Symbia Intevo 16 |
| ECG-gating | No | No | No | No |
| Breath-holding | Yes (end expiratory) | No | No | Yes (end-expiratory) |
| Slice Thickness [mm] | 5.0 | 2.5 | 3.0 | 5.0 |
| Tube Current [mA] | 16 | 60/150* | 30 | 20 |
| Tube Voltage [kVp] | 120 | 120 | 120 | 120 |

*acquisition parameters adjusted for patients with body mass index ≥ 40 kg/m^2^

**Supplementary Table 9**. Selected organs, radiomics features and MPI parameters

| Organs (n=33) | First-Order Features^‡^ (n=11) | 3D Shape Features^‡^ (n=4) |
| --- | --- | --- |
| Myocardium | 10^th^ Percentile | Elongation |
| Left Atrium | 90^th^ Percentile | Flatness |
| Left Ventricle | Kurtosis | Sphericity |
| Right Atrium | Maximum | Voxel Volume |
| Right Ventricle | Minimum |  |
| Left Atrial Appendage | Mean |  |
| Aorta | Robust Mean Absolute Deviation |  |
| Pulmonary Artery | Root Mean Squared |  |
| Lungs^**^  Trachea | Skewness  Total Energy |  |
| Esophagus | Uniformity |  |
| Stomach |  |  |
| Liver |  |  |
| Spleen |  |  |
| Pancreas |  |  |
| Adrenal Glands^*^ |  |  |
| 4^th^ - 12^th^ Thoracic Vertebrae |  |  |
| Erector Spinae Muscle^*^ |  |  |
| Spinal cord |  |  |

^*^ right and left, ^**^ right upper lobe, right middle lobe, right lower lobe, left upper lobe, left lower lobe

^‡^ Radiomics

MPI – myocardial perfusion imaging

**Supplementary Table 10.** Comparison between models based on all 32 radiomic features from first-order statistics and 3D shape-based categories, and a subset of 15 clinically interpretable features.

|  | 32 vs. 15 radiomic features | |  |
| --- | --- | --- | --- |
| Model | 32 features | 15 features | P-value (DeLong) |
| AIl | 0.80 [0.74, 0.85] | 0.80 [0.74, 0.87] | 0.09 |
| AI hybrid | 0.78 [0.73, 0.84] | 0.79 [0.72, 0.86] | 0.01 |
| AI CTAC | 0.78 [0.72, 0.83] | 0.78 [0.71, 0.85] | 0.40 |

Values are presented as mean [95% confidence interval]

ACM - all-cause mortality; AI - artificial intelligence; AUC - area under receiver operating characteristic; CTAC – computed tomography attenuation correction.

**Supplementary Table 11.** SPECT imaging features

|  | Features |
| --- | --- |
| Stress | Total Perfusion Deficit |
|  | Quality control |
|  | Volume |
|  | Shape index, end-diastolic |
|  | Shape index, end-systolic |
|  | Length |
| Stress gated | Ejection fraction |
|  | Volume, end-diastolic |
|  | Quality control |
|  | Shape index, end-diastolic |
|  | Shape index, end-systolic |
|  | Length, end-diastolic  Length, end-systolic |
|  | Motion extent for all segments |
|  | Motion raw for all segments |
|  | Thickening extent for all segments |
|  | Thickening raw for all segments |
|  | Wall volume, end-diastolic |
|  | Wall volume, end-systolic |
|  | Volume, end-systolic |
|  | Bandwidth for the entire ventricular counts in the segment |
| Rest | Total perfusion Deficit |

SPECT – single-photon emission computed tomography
